# Evaluation of Morphological and Immunohistochemical Prognostic Parameters in Giant Cell Tumor of Bone*

**DOI:** 10.5146/tjpath.2026.14827

**Published:** 2026-05-30

**Authors:** Cem Berk Türk, Hüseyin Kemal Türköz, Ömer Sofulu, Bülent Erol

**Affiliations:** Department of Pathology, Marmara University School of Medicine, İstanbul,Türkiye;Marmara University, Pendik Training and Research Hospital, İstanbul,Türkiye; Department of Orthopedics and Traumatology, Marmara University School of Medicine,İstanbul,Türkiye;Marmara University, Pendik Training and Research Hospital,İstanbul,Türkiye

**Keywords:** Giant cell tumor of bone, Prognosis, p63, RANKL, VEGF

## Abstract

Objective: Giant cell tumor of bone (GCTB) is a rarely metastasizing, locally aggressive tumor. Recurrence and metastasis in GCTB are thought to be driven by the biological behavior of the neoplastic stromal mononuclear cells. Several pathophysiological mechanisms, including expressions of p63, RANK, RANKL, and VEGF, have been proposed to influence this aggressive potential. This study aimed to identify histomorphological and immunohistochemical parameters that may predict recurrence and metastasis in patients with GCTB.

Material and Methods: This retrospective study included 32 GCTB patients. Clinical, radiological, and pathological data were reviewed. Histomorphological features, including surgical margins, bone cortex invasion, soft tissue invasion, mitotic count, vascular invasion, percentage of spindle cell areas, and presence of tumor-associated lymphocytes (TALs), along with tumor size and demographic features, were evaluated. Tissue sections were stained with p63, RANK, RANKL, and VEGF. The relationship between these parameters and post-surgical recurrence or metastasis was analyzed.

Results: A higher percentage of spindled pattern was significantly associated with a lower frequency of recurrence. A larger tumor diameter at diagnosis was associated with the development of metastasis. Other histomorphological parameters and the expression of p63, RANK, RANKL, and VEGF were not significantly associated with recurrence or metastasis.

Conclusion: Percentage of spindled pattern and primary tumor diameter are potential prognostic factors for recurrence and metastasis, respectively, in GCTB.

## Introduction

Giant cell tumor of bone (GCTB) is a locally aggressive neoplasm that rarely metastasizes (1). GCTB typically affects the epiphyses of long bones in the mature skeleton and constitutes 4-5% of all primary bone tumors. It is most commonly observed between ages 20 and 45 (2). More than 90% of GCTBs have a mutation at position G34 in the H3F3A gene, which encodes the histone variant H3.3. The most common mutation is G34W, where the amino acid Glycine (G) at position 34 of the H3.3 histone is changed to Tryptophan (W) (3,4). The H3.3G34W mutation can be demonstrated immunohistochemically with a specific antibody that marks neoplastic mononuclear cells (5,6). GCTB is usually treated with curettage. Local adjuvant therapy can be applied with agents such as phenol, liquid nitrogen, and hydrogen peroxide (7). En-bloc resection is an option for tumors with frequent recurrences or for locations where resection does not significantly alter function (e.g., fibula) (2,6). Tumors recur locally in 15-50% of patients, mostly within 2 years after curettage. It can metastasize to the lungs in 3-7% of patients (2).

Recurrence or metastasis in GCTB cases has been associated with histomorphological and clonal characteristics of mononuclear cells (8–11). However, no clearly defined histomorphological features associated with recurrence and metastasis have been reported to date.

p63 protein is expressed in a subset of the mononuclear cells in GCTBs and is considered to constitute the group of cells that transform into osteoblasts (12). It has been reported that the mean p63 expression rate in recurrent GCTB cases was significantly higher compared to non-recurrent cases (9).

Overexpression of RANKL by neoplastic mononuclear cells stimulates RANK-expressing monocytic cells to form osteoclastic giant cells. This increased RANK/RANKL activity leads to increased bone matrix erosion and release of growth factors from the matrix that stimulate cell proliferation in GCTB. Ultimately, this cycle of increased bone remodeling and cell proliferation may increase the risk of recurrence and metastasis (10,11,13).

VEGF is a growth factor and an important regulator involved in angiogenesis (14). GCTBs are hypervascularized tumors, and there are opinions that VEGF expression increases in cases showing a clinically aggressive course. It was suggested that VEGF was responsible not only for angiogenesis but also for the migration of mononuclear cells within GCTB. Evaluating the relationship between VEGF and GCTB aggressiveness may aid early detection of metastases (14–16).

As a general principle in tumor immunology, tumor-associated lymphocytes (TAL) may respond inappropriately to conventional stimuli or not respond at all (17–19). It was reported that the inflammatory tumor microenvironment promoted angiogenesis, tumor proliferation, and metastasis (20).

We aimed to evaluate the pathophysiological mechanisms described above in GCTB cases with and without recurrence/metastasis, and to identify histomorphological and immunohistochemical parameters that predict the risk of recurrence/metastasis.

## Materials and Methods

This study retrospectively included 38 cases diagnosed as GCTB in the records of Marmara University Pendik Training and Research Hospital, Department of Pathology between 01.01.2012 and 31.10.2023. Inclusion criteria for the study included the presence of a GCTB diagnosis in the pathology laboratory records, clinical follow-up of the cases, presence of paraffin blocks, and sufficient tissue in the blocks for additional examinations. Cases that did not show a diffuse staining pattern with the H3.3G34W marker were excluded, and a total of 32 cases were included in the study.

During the pathological evaluation of cases, H3.3G34W immunohistochemistry-stained slides from the archive were examined; H3.3G34W staining was also applied to cases for which no slide was available for this marker. After determining the areas where the tumor was best represented, immunohistochemical examination was performed by applying RANK, RANKL, p63, and VEGF markers.

For all IHC protocols (Table 1), 2 µm thick sections taken from formalin-fixed paraffin-embedded tissues were placed on electrostatically charged slides and dried at 72°C for 1 hour. All procedures, including deparaffinization and antigen retrieval, were performed using the ROCHE/Ventana BenchMark XT IHC Stainer or Ventana BenchMark Ultra automation systems. A ready-made kit containing biotin-free, HRP (horseradish peroxidase) multimer-based, hydrogen peroxide substrate, and 3,3’-diaminobenzidine tetrahydroxychloride (DAB) chromogen (UltraviewTM, Universal DAB Detection Kit, Catalog Number 760-500, Ventana Medical Systems, Tucson, AZ) was used for the procedure. The process was finalized by manually performing counterstaining with hematoxylin and bluing reagent, dehydrating the sections in the staining device, clearing with xylene, and covering with a coverslip.

**Figure 1:**
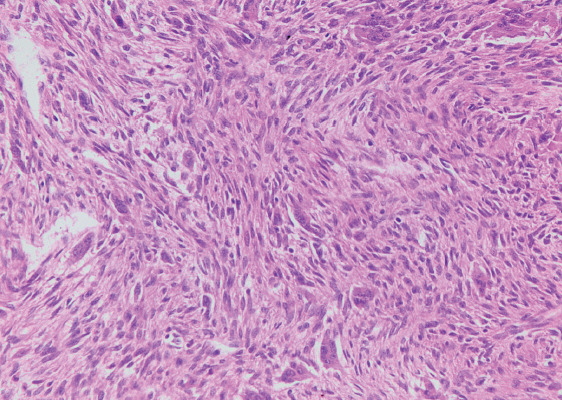
1: : Spindle cell morphologic pattern

**Table 1:** Summary of antibodies and protocols used for immunohistochemistry.

**Antibody**	**Clone**	**Dilution**	**Incubation Time**	**Antigen Retrieval**	**Detection System**
VEGF	SC-53462	1:50	120 min	Leica ER2 (EDTA), 40 min	DAB
RANK	SC-52951	1:25	120 min	Roche CC1 (EDTA), 90 min	DAB
RANKL	PA5-20291	1:100	120 min	Roche CC1 (EDTA), 90 min	DAB
p63	EP174	1:100	60 min	Roche CC1 (EDTA), 60 min	DAB
H3.3 G34W	RM263	1:100	60 min	Roche UltraView Universal DAB	DAB

H&E-stained slides were evaluated for surgical margin, bone cortex invasion, soft tissue invasion, total number of mitoses in 10 high-power fields (HPF) at the hot-spot, vascular invasion, percentage of spindle cell areas (Figure 1), and presence of TALs. The assessment of vascular invasion was made with consideration of the possibility of false positives due to the curettage method used to obtain the tissue samples. Only frank endothelial invasion through the vascular lumen and tumor thrombus in the vascular lumen were counted as vascular invasion. TALs were quantified by summing the total number of lymphocytes in 10 HPFs, selected from the most densely infiltrated areas among neoplastic cells. Hemorrhage areas were excluded to prevent possible misinterpretations.

Bone cortex and soft tissue invasion were determined based on radiological and/or histopathological findings. Any disruption of cortical bone integrity by a tumor was considered cortical invasion. Tumor cells that make frankly invasive clusters within connective tissue were considered as soft tissue invasion. Radiological evidence of surrounding soft tissue invasion was also regarded as soft tissue invasion.

p63 staining was evaluated by manually counting positive neoplastic mononuclear cells as a percentage of the total neoplastic mononuclear cell population in 10 HPFs at hot-spot areas. Neoplastic cells in the highest H3.3G34W-stained regions that were chosen for immunohistochemical evaluation were carefully identified and distinguished from the reactive non-neoplastic cells based on their distinct morphology.

**Figure 2:**
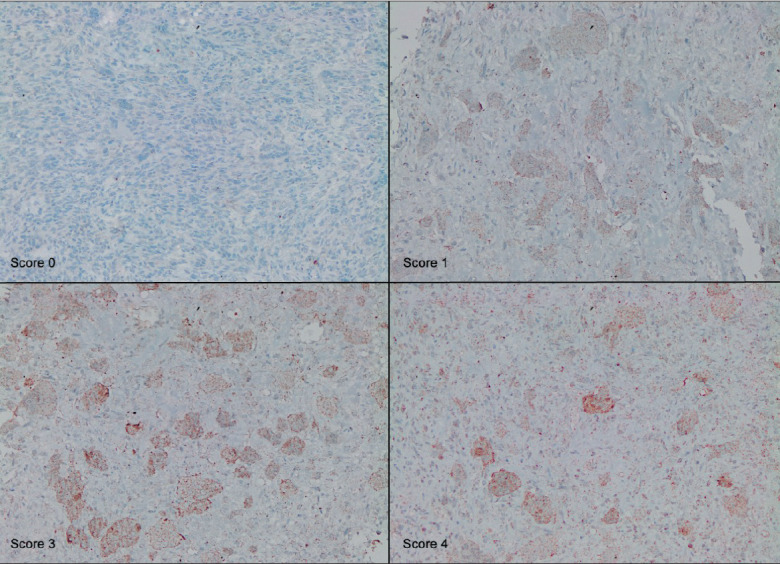
RANK expression at varying intensities

**Figure 3:**
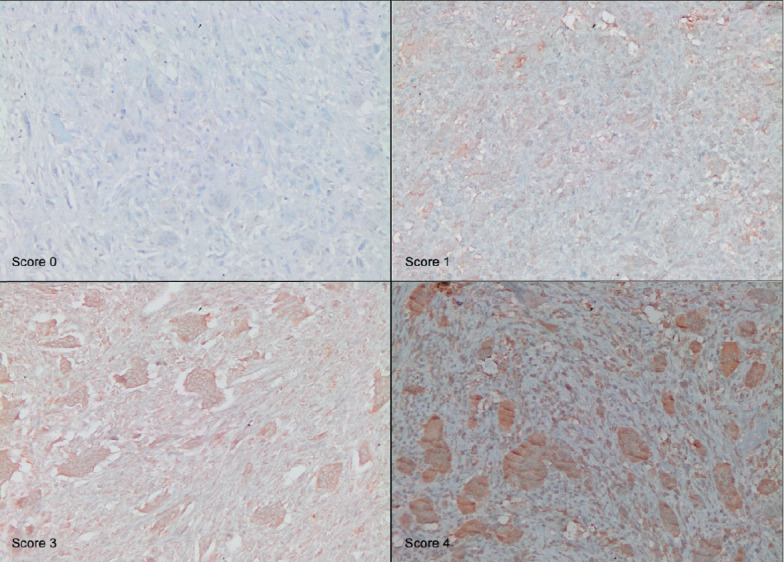
RANKL expression at varying intensities

Regarding high variability in staining density of RANK and RANKL antibodies, the strength of staining was scored between 0 and 3 (0: none, 1: weak, 2: moderate, 3: strong). The percentages of staining for each strength level were calculated. Each staining strength score was multiplied by the percentage of staining at that strength level to express staining intensity. The results were summed to determine the final RANK (Figure 2) and RANKL (Figure 3) scores.

For VEGF IHC examination, cytoplasmic staining in neoplastic mononuclear cells was evaluated. The percentage of cytoplasmic staining in mononuclear cells was determined by visual estimation across the entire tumor area of the tissue section.

The IBM Statistical Package for the Social Sciences (IBM SPSS version 26.0) program was used for statistical analysis to evaluate the findings obtained in the study. Descriptive analyses were calculated with median values for continuous data when they did not show normal distribution, and with numerical and percentage values for categorical data. The Shapiro-Wilk test was performed to determine whether the continuous data were suitable for a normal distribution. The Mann-Whitney U test was used as the continuous data did not conform to the normal distribution. Fisher’s Exact Test was used in pairs for the comparison of categorical data. Spearman’s Rank Correlation Test was used in pairs for the comparison of continuous data. Cox regression analysis method was used for disease-free survival analysis and multivariate analysis. A value of p<0.05 was accepted for statistical significance.

## Results

The study included 32 cases of GCTB; 11 were male, and 21 were female, with a mean age of 40 years. 29 tumors were located in long bones, while 3 affected short or flat bones. The average tumor diameter at the time of diagnosis was 6.2 cm. The largest diameter was 18 cm, and the smallest was 2.4 cm.

The initial diagnostic biopsy was a curettage in all cases except 1 case. Subsequently, en bloc resection was performed in 2 cases (18.1%), and excisional biopsy in 2 cases (18.1%). Patients did not get any additional medical therapy.

Cases that received a GCTB diagnosis at the exact tumor location at least 1 month after the initial diagnosis were considered as recurrence. Clinical, radiological, and/or pathological recurrence was detected in 11 (34.3%) of the 32 cases during follow-up. Among the recurrent cases, the mean time until recurrence was 16 months. The shortest period was 1 month, and the longest was 60 months. The disease recurred only once, in one case. Distant metastasis was observed in 3 cases (9.3%). The location of distant metastasis was the lung in all three cases. Two of the metastatic cases also showed recurrence. In cases with distant metastasis, the interval between resection and metastasis ranged from 16 to 135 months, with a median of 25.3 months. When all cases were considered together, the mean disease-free survival time was 51.5 months.

No significant relationship was found between recurrence/metastasis and gender, age, tumor location, or surgical method. When tumor diameter, mitosis count, spindle cell ratio, TALs, bone cortex, and soft tissue invasion were compared with recurrence and metastasis, a significant relationship was found between the tumor diameter at the time of diagnosis and metastasis, with the risk of metastasis increasing as the tumor diameter increased (Table 2). The spindle cell pattern ratio was evaluated as the percentage of spindle cell pattern areas observed in the tumor parenchyma, and the average ratio was 27.8%. The analysis showed that a higher spindle pattern ratio was related to a lower risk of recurrence. Recurrence was more frequent, especially in cases with a spindle pattern percentage below 20% (95% CI: 20.2-44.7). It was found that an increase in the spindle pattern percentage reduced the probability of recurrence by 1.3 times. There was no significant relationship between the ratio of spindle cell pattern and metastasis. No statistically significant relationship was detected in other parameters (Table 2).

**Table 2:** Comparison of tumor diameter, mitosis count, spindle cell percentage, and TALs by recurrence and metastasis

-	**Recurrence**		**Metastasis**	
**Parameters (mean)**	**Absent**	**Present**	**Absent**	**Present**
Tumor Diameter	6.59	5.54	5.95	8.90
	p = 0.858		p = 0.044*	
Mitosis Count	3.95	3.82	4.07	2.33
	p = 0.855		p = 0.842	
Spindle Cell Percentage	34.76	14.55	25.17	53.33
	p = 0.044*		p = 0,254	
TALs	163.50	135.73	158.69	80.50
	p = 0.750		p = 0.154	

*p < 0.05

Associations of bone cortex, soft tissue, and vascular invasion with recurrence and metastasis are summarized in Table 3. Statistical analysis revealed no significant relationship among these variables.

**Table 3:** Comparison of cortical bone, soft tissue, and vascular invasion by recurrence and metastasis

-	-	**Recurrence (n)**		**Metastasis (n)**		**Mean DFS (Months)**
**Parameters**	**Status**	**Absent**	**Present**	**Absent**	**Present**	
Cortical Bone Invasion	Absent	5	1	5	1	44.8
	Present	15	10	23	2	54.0
	-	p = 0.382		p = 0.488		-
Soft Tissue Invasion	Absent	10	2	12	0	66.4
	Present	9	8	14	3	40.6
	-	p = 0.126		p = 0.246		-
Vascular Invasion	Absent	15	8	20	3	56.0
	Present	6	3	9	0	39.9
	-	p = 1		p = 0.540		-

Evaluation of surgical margins was limited because tissues were obtained by curettage, and materials were highly fragmented in most cases. No recurrence was observed among cases that underwent en bloc resection. The number of cases was insufficient to evaluate the significance of surgical margins statistically.

When immunohistochemical markers were evaluated, no statistically significant associations were found between p63, VEGF, RANK, and RANKL and recurrence or metastasis (Table 4). Comparison of demographic and clinical data against immunohistochemical and histomorphological findings revealed no statistically significant relationships.

**Table 4:** Comparison of immunohistochemical markers by recurrence and metastasis

-	**Recurrence**		**Metastasis**	
**Parameters (mean)**	**Absent**	**Present**	**Absent**	**Present**
p63	37.90	46.01	42.58	22.33
	p = 0.226		p = 0.128	
VEGF	5.81	5.64	6.34	0.00
	p = 0.708		p = 0.072	
RANK Score	118.10	169.09	143.45	60.00
	p = 0.188		p = 0.217	
RANKL Score	116.90	162.73	130.00	158.33
	p = 0.437		p = 0.896	

A significant relationship was observed between spindle pattern percentage and the RANK marker (p=0.03). It was determined that as the spindle pattern percentage increased, the staining rate with the RANK marker decreased. No statistically significant relationship was detected between the other immunohistochemical markers and the assessed histomorphological features.

Comparing immunohistochemical markers with each other showed that the RANK marker staining rate significantly correlated with the staining rates of both RANKL (p=0.02) and p63 (p=0.03). Higher staining amounts for the RANK marker were associated with increased staining for p63 and RANKL. No significant relationship was found in the comparison of other markers.

In the multivariate analysis, no parameter was identified as an independent risk factor for recurrence.

## Discussion

In our cohort, the overall recurrence rate was 11 (34.3%), consistent with the medical literature (2,21,22). There were only 3 cases with metastasis. We did not find any association between recurrence and metastasis and demographic features such as age and gender (2,21–24). No significant association was found between tumor location and recurrence, metastasis, or disease-free survival, consistent with previous studies (22,25).

Curettage was the most common treatment in our cases, accounting for 96.8% (31 cases). This approach parallels current literature data (2,22,26). Due to the high frequency of curettage in our cohort, the association between surgical excision method and recurrence or metastasis could not be evaluated. Consequently, surgical margin assessment could not be performed. Studies with a higher number of en bloc resections would be useful for this purpose.

Although tumor size is often cited as a risk factor for recurrence (2,8,27), our analysis revealed no statistically significant association between tumor size and recurrence. This finding mirrors that of Zhou et al. (28), who also reported no significant association between tumor size and recurrence. However, we observed a significant association between tumor diameter at diagnosis and distant metastasis; larger tumors were more likely to metastasize. This finding suggests that the large diameter may reflect aggressive tumor behavior, including metastatic potential.

We observed high rates of bone cortex invasion and soft tissue invasion. However, neither factor showed a statistically significant association with recurrence/metastasis in our study. This finding contrasts with a conflicted literature: Zhou et al. (28) linked cortical thinning, but not soft tissue invasion, to recurrence, whereas Balke et al. (21) and Chan et al. (23) found that both parameters were significantly associated with recurrence and metastasis. Additional studies offer mixed results (15,22,29). The high frequency of both cortical bone and soft-tissue invasion in our patients without recurrence/metastasis likely explains the lack of a statistically significant association between these variables in our study.

We observed an average spindle pattern ratio of 27.8%, and found that recurrence frequency increased 1.3 times in cases with a spindle pattern below 20% (p=0.04). Although there are studies suggesting spindle cells are associated with reactive bone formation and collagen synthesis (30,31), there is no study examining the percentage of spindle pattern as a recurrence risk factor. Our findings imply that a higher spindle cell component may facilitate total resection by limiting tumor invasion with desmoplasia-related stromal changes. Although the relationship between spindle cell percentage and recurrence was not found to be an independent variable in the multivariate analysis (p=0.08), it was close to the significance threshold.

In the study by Quattrini et al., the frequency of metastasis was higher in cases with high RANK and RANKL expression. In the same study, no relationship was defined between recurrence and RANK and RANKL expression (10). RANK and RANKL expression did not correlate with recurrence and/or metastasis in our study. Our findings suggest that, despite the well-known effects of RANK and RANKL on bone matrix destruction, their expression levels in tumor cells do not predict recurrence or metastasis. Our statistical analyses revealed significant relationships between RANK expression and RANKL expression levels, as well as between RANK expression and p63 expression levels. High RANKL score in cases with high RANK score is expected and can be attributed to the receptor-ligand relationship of these molecules. We found higher RANK levels with increasing p63 levels. This correlation suggests that p63 stimulates neoplastic cells to show osteoblastic activity, as previously reported (12). We have also observed a significant negative correlation between the RANK score and the ratio of the spindle cell pattern. It was observed that RANK scores decreased as the spindle pattern percentage increased. This finding suggests that the spindle cell pattern is associated with desmoplastic host response; therefore, as the proportion of the spindle cell pattern increases, RANK expression, osteoclastic activity, and recurrence rate decrease.

A positive correlation was found between tumor diameter at the time of diagnosis and the frequency of metastasis, suggesting that tumors with larger sizes are biologically more aggressive and metastasize more frequently. Additionally, a negative correlation was observed between the spindle pattern ratio and both RANK score and tumor recurrence rate, suggesting that the spindle pattern may be associated with a tumor-limiting desmoplastic response mechanism and can be evaluated as a prognostic parameter.

GCTB is a rare tumor and exhibits known local aggressive behavior. However, its recurrence and metastatic potential cannot yet be clearly estimated. The limited sample size of our study may reduce the statistical power of the results. Furthermore, previous studies evaluating prognostic parameters for estimating the biological behavior of this condition have yielded inconsistent or conflicting findings. These combined limitations underscore the necessity for larger, multicenter series to comprehensively evaluate these parameters and establish definitive predictive factors for the biological behavior of GCTB.

### Conflict of Interest

The authors report there are no competing interests to declare.

### Funding

This study was financially supported by the Marmara University Scientific Research Project Unit (Project ID: 10914, 12.03.2023)

### Ethical Approval

This study, approved by the Clinical Research Ethics Committee of Marmara University (issue date 16/10/2022, number 09.2022.1133), was conducted in accordance with the 1964 Helsinki declaration and its later amend-ments or comparable ethical standards.
